# Seasonal temperatures in South Eleuthera, The Bahamas, have considerable impacts on the cardiorespiratory function and swimming performance of Nassau grouper (*Epinephelus striatus*)

**DOI:** 10.1093/conphys/coad086

**Published:** 2023-12-05

**Authors:** E S Porter, A K Gamperl

**Affiliations:** Department of Ocean Sciences, Memorial University of Newfoundland and Labrador, 0 Marine Lab Rd., St. John’s, NL A1C 5S7, Canada; Department of Ocean Sciences, Memorial University of Newfoundland and Labrador, 0 Marine Lab Rd., St. John’s, NL A1C 5S7, Canada

**Keywords:** Cardiac function, exercise, grouper, metabolism, oxygen extraction, temperature

## Abstract

Surprisingly, the impacts of environmental changes on the physiology of tropical/subtropical marine fishes have received limited attention. Given that (i) temperature is considered to be a key factor controlling the biology of fishes; (ii) no published data are available on the swimming performance, metabolic capacity or cardiac function of any of the ~165 grouper species worldwide; and (iii) the Nassau grouper is an endangered species of great ecological and socioeconomic significance in The Bahamas, we investigated how current summer/early fall (30°C) and winter (22°C) temperatures in South Eleuthera affected the aerobic metabolism and heart function of wild Nassau grouper when swum to exhaustion (i.e. to their critical swimming speed, *U*_crit_). The Nassau grouper had a very low *U*_crit_ at 30°C (i.e. <1 body lengths s^−1^), and a 30% lower swimming performance during the winter (at 22°C), and this was that was indicative of a reduced absolute aerobic scope (~185 vs. 290 mg O_2_ kg^−1^ h^−1^) and values of maximum heart rate ($f$_HMax_) and scope for $f$_H_ that were only one-half of that achieved at 30°C (~60 vs. 120 and 29 vs. 61 beats min^−1^, respectively). Overall, these data reveal that the Nassau grouper’s aerobic and swimming capacity are well below values reported for other tropical/subtropical fishes and suggest that, despite a compensatory (~30–40%) increase in stroke volume, constraints on $f$_H_ near this species’ lower thermal limit negatively affect its cardiac output and swimming performance. These findings have considerable ecological implications as Bahamian grouper populations migrate over long distances to spawn during the winter months, and given the predicted increase in temperature variability with climate change.

## Teaser

Nassau grouper are important for fisheries and reef ecosystem stability in The Bahamas, however, their populations have been declining for decades. We report that this species’ swimming and aerobic capacity, and maximum cardiac function, are quite limited and reduced by 30 – 40% at winter temperatures (22oC) vs. in the summer/early fall (30oC).

## Introduction

Groupers (subfamily Epinephelinae) are a phylogenetically diverse group of fishes composed of ~165 species that are distributed widely throughout tropical and subtropical oceans (IUCN, 2011; Ma *et al.,* 2016). They are predatory fishes of great importance for reef ecosystem stability and in commercial and recreational fisheries, particularly in Asian and Caribbean countries (FAO, 2014 and 2022; Ma *et al.,* 2016; Sadovy de Mitcheson *et al.,* 2013). However, without regular monitoring, they have become increasingly vulnerable to fishing pressures and overexploitation, given their unique life history traits, including late sexual maturation and aggregation spawning (Colin, 1992; Sala *et al.,* 2001; Sadovy de Mitcheson *et al.,* 2013; Sherman *et al.,* 2016). While there have been efforts to establish more marine protected areas (MPAs), drastic reductions in the population size of several species have been observed (i.e. decreases in spawning aggregation sites as well as in the number of individuals at each site), and this suggests that the restoration of wild stocks will require more effective long-term conservation and management strategies (Golbuu and Friedlander, 2011; Andrello *et al.,* 2013; Anderson *et al.,* 2014).

Although knowledge of grouper biology is increasing, the current literature for this taxon is limited to several topics, including their life history traits [i.e. migration (Bolden, 2000; Starr *et al.,* 2007) and spawning (Colin, 1992; IUCN, 2018; Sala *et al.*, 2001)], population dynamics (Sherman *et al.,* 2020) and the ecological importance they hold for maintaining reef systems (Eggleston *et al.,* 1998; Harmelin and Harmelin-Vivien, 1999). Considering that (i) temperature has a major influence on the biology and physiology of aquatic ectotherms (Brett, 1971) and (ii) average ocean temperatures are predicted to rise by 2–4°C by 2100 (Cooley *et al.,* 2022), it is not surprising that ecophysiologists are examining the impacts of temperature changes on the biology of important marine species, including fishes. Aerobic scope (the amount of oxygen consumption/aerobic-based metabolism available above that required to support essential physiological functions) and thermal performance curves have been extensively used to estimate a species’ sensitivity to temperature change (Fry, 1971; Pörtner, 2001; Pörtner and Knust, 2007; Schulte *et al.,* 2011). However, there are several other factors that are central to understanding a fish’s performance at a given temperature, including cardiac function [heart rate (*f*_H_), cardiac output ($\dot{Q}$) and stroke volume (*V*_S_)], blood oxygen-carrying capacity and tissue oxygen extraction ($\dot{\mathrm{M}}$O_2_/$\dot{Q}$), and muscle contractility and contraction kinetics (Gollock *et al.,* 2006; Harter *et al.,* 2019; Gamperl and Syme, 2021; Leeuwis *et al.,* 2021; Ern *et al.,* 2023). The majority of research in these areas has focused on the impacts of different thermal/acclimatory environments on a few commercially and recreationally relevant cold water fishes, including salmonids [Arctic char (*Salvelinus alpinus*), rainbow trout (*Oncorhynchus mykiss*) and Atlantic salmon (*Salmo salar*)], Atlantic cod (*Gadus morhua*) and European perch (*Perca fluviatilis*) (Gollock *et al.,* 2006; Penney *et al.,* 2014; Sandblom *et al.,* 2016; Motyka *et al.,* 2017; Gilbert and Farrell, 2021), and little is known about the *in vivo* cardiorespiratory physiology of subtropical/tropical marine fishes (Nelson *et al.,* 2017; Heuer *et al.,* 2021; Morgenroth *et al.,* 2022; Schneider *et al.,* 2023). Furthermore, although research suggests that tropical reef fishes have a narrow thermal window (i.e. the difference between the minimum and maximum temperatures that they can tolerate) and are currently living at temperatures approaching their upper thermal limits (Farrell, 2009; Nilsson *et al.,* 2009; Rummer *et al.,* 2014), there are no published data on the metabolic/aerobic capacity, swimming performance or cardiac function of any of the ~165 grouper species.

The Nassau grouper (*Epinephelus striatus*) is a subtropical species that inhabits shallow coral reefs and rock formations in the western Atlantic Ocean and Caribbean Sea (Domeier and Colin, 1997; Starr *et al.,* 2007). Their migrations to reproduce in large spawning aggregations at specific times and locations have resulted in the overfishing of many wild Caribbean stocks (Colin, 1992; Sala *et al.,* 2001; Starr *et al.,* 2007). For example, even with the establishment of a moratorium in 2004 on fishing during the spawning season, commercial landings of Nassau grouper in the Bahamas continued to fall and declined by approximately one-half between 2006 and 2014 (Sherman *et al.,* 2016), and in 2016, they were officially listed as threatened under the US Endangered Species Act (Federal Register, 2016). Given the ecological and socioeconomic importance of the Nassau grouper, the status of current populations of this species and the major challenges that climate change poses to tropical marine fishes, it is clear that information on the temperature-dependent biology and physiology of this species is required to support future conservation and management efforts.

As a starting point, the main goal of this study was to measure the swimming performance, metabolic capacity and cardiac function of wild-caught Nassau grouper at temperatures that approximate the lower (22°C) and upper (30°C) temperatures that subadults of this species would normally experience at shallow patch reefs in The Bahamas (e.g. see Colin, 1992; Schneider *et al.,* 2023; and Supplementary Figure S1). However, these temperatures were also chosen: (i) as winter is the time of year that Nassau grouper in the Bahamas migrate considerable distances to spawn, and measurements at temperatures typical of this season would allow us to estimate their metabolic and swimming capacity at this critical time in their life cycle (Starr *et al.,* 2007); and (ii) as they would allow us to further examine the hypothesis put forward by Gamperl *et al.* (2022). These authors predicted that because myocardial contraction/twitch kinetics greatly constrain maximal heart rate (*f*_HMax_) at cool/cold temperatures, stroke volume (*V*_S_) would play a greater role in meeting the cardiac pumping demands of exercise at these temperatures. This hypothesis is supported by recent work on Atlantic salmon (*S. salar*; Porter and Gamperl, 2023), but data on other species, and with different thermal niches, are needed to establish whether this is a universal phenomenon.

## Materials and Methods

This study was approved by the Animal Care Committee of Memorial University of Newfoundland and Labrador (protocol no. 22-01-KG) and by The Bahamas’ Departments of Marine Resources and Environmental Protection and Planning (DEPP Research Permit No. BS-2022-873 637). All procedures conducted on Nassau grouper were performed in accordance with the Canadian Council on Animal Care’s Guidelines on the ‘Care and Use of Fish in Research, Teaching and Testing’ (Canadian Council on Animal Care, 2005).

### Experimental animals

Wild subadult Nassau grouper (see Supplementary Table S1) were caught on shallow reefs (~5–12 m in depth) near the Island School’s Cape Eleuthera Institute (CEI) in South Eleuthera using rectangular baited traps in the early fall (12 October to 15 November 2022) and winter (25 February to 25 March 2023). After slowly being brought to the surface, the fish were transported in 65 l coolers containing aerated seawater by boat to the CEI wet lab. Fish were held outdoors in 1.3 m^3^ cylindrical tanks under natural photoperiod for no longer than 2 weeks prior to experimentation. Tanks were supplied with flow-through seawater (~34 ppt and >95% air saturation) with temperatures (± SD) averaging 27.2 ± 1.4°C (range 23.8–29.2°C) and 24.2 ± 1.4°C (range 21.5–26.8°C) in the early fall and winter, respectively. Fish were fed sardines (*Sardinella aurita*) to satiation three to four times per week and were fasted for 24–48 h prior to surgery.

### Surgical procedures

Fish were netted from their holding tank and anaesthetized in aerated seawater containing tricaine methanesulfonate (TMS, 0.2 g l^−1^; Syndel Laboratories Ltd, Qualicum Beach, BC, Canada) until ventilatory movements ceased. Weight (grams), fork length (centimetres) and girth (centimetres) were measured, and the fish were placed on their right side on a wetted foam pad upon a surgical table where their gills were continuously irrigated with aerated seawater containing a maintenance dose of TMS (0.1 g l^−1^) at tank temperature. A Transonic^®^ flow probe (2.0–2.5 PSS; Transonic Systems Inc., Ithaca, NY, USA) was fitted around the ventral aorta (Gollock *et al.,* 2006; Leeuwis *et al.,* 2021) and connected to a Transonic^®^ flow meter (model T402-A20029; Transonic Systems Inc.) to ensure the signal was of high quality. The probe lead was then secured to the fish at 4 locations using 2–0 silk suture: to the ceratobranchial element lining the posterior margin of the fourth buccal-opercular opening, which is absent of gill filaments; just posterior to the pectoral fin; just below the lateral line; and just anterior to the dorsal fin (e.g. see Supplementary Figure S1 in Leeuwis *et al.,* 2021).

### Critical swim speed (*U*_crit_) test

After surgery was completed, each fish (*n* = 9 per temperature) was transferred to a 108.7-l Blazka-type swim-tunnel respirometer with an internal diameter of 24.0 cm and a 100-cm-long working section that was filled with ambient seawater (i.e. at the same temperature as the holding tank). The front of the respirometer was fitted with a plastic grid, which ensured uniform water flow in the swimming section of the respirometer (Taylor and McPhail, 1985), and was covered with black plastic to provide the fish with a dark refuge and to minimize stress from external stimuli. The impellor was powered by a Leeson Washguard three-phase AC motor (model C182T17WK3D; Leeson Electric, Grafton, Wisconsin, USA) and controlled by a Leeson Speedmaster motor controller (model 174 526, 0–120 Hz, Leeson Electric). Seawater was supplied to the swim tunnel from a temperature-controlled 208-l reservoir at 10 l min^−1^, and the O_2_ content of the water was maintained at >95% air saturation by bubbling the reservoir with air. At ~6 h post-surgery, temperature in the reservoir was either increased or decreased at 1°C h^−1^ to the desired test temperature (30 or 22°C) using a 1800-W programmable immersion heater (Intelligent Heaters, GA, USA) or a ½-hp Drop-In chiller (Aqualogics Inc., NC, USA), respectively. These temperature changes represented increases and decreases of 2.16 ± 0.21 and 1.08 ± 0.36°C, respectively, as compared to when the fish were netted from the tanks. Then, at ~8–10 h post-surgery, a brief (2- to 5-min) ‘training session’ was performed, during which the water velocity was gradually increased to ~0.5 body lengths s^−1^ (bL s^−1^). Once the fish swam for several minutes, the velocity was brought back down to 0.08 bL s^−1^ and the fish was left to recover overnight (i.e. for 12–14 h).

Approximately 24 h after surgery, a critical swim speed (*U*_crit_; Brett, 1964) test was used to measure resting, active/maximum and post-exhaustion cardiac and metabolic parameters. Resting values for parameters of cardiac function (see below) and oxygen consumption ($\dot{\mathrm{M}}$O_2_; in milligrams O_2_ kg^−1^ h^−1^) were measured at the baseline speed (0.08 bL s^−1^). Then, swimming velocity was slowly increased to 0.35 bL s^−1^ (the first speed that the fish would continuously swim), and this was followed by velocity increments of 0.15 bL s^−1^ every 15 min until the fish reached exhaustion (i.e. the inability of the fish to move away from/off the back grid for >10 s). At this point, current velocity was immediately reduced back to the baseline level for 2 h, during which cardiac parameters and $\dot{\mathrm{M}}$O_2_ were measured every 15 and 30 min, respectively. The fish’s *U*_crit_ was calculated as (Brett, 1964):


(1) \begin{equation*} {U}_{crit}=V+\left[\left({t}_f\times \ {V}_i\right) /\ {t}_i\right] \end{equation*}

where *V* = velocity at which the fish swam for the entire time increment; *V*_i_ = velocity increment (0.15 bL s^−1^); *t*_f_ = time elapsed from the last change in current velocity to fatigue; and *t*_i_ = time increment (i.e. the time between increases in velocity; 15 min). Then, *U*_crit_ was corrected for the solid blocking effect of the fish (Bell and Terhune, 1970; Kline *et al.,* 2015) using the formula:


(2) \begin{equation*} {V}_F={V}_R\left(1+{\in}_S\right) \end{equation*}

where *V_F_* was the water velocity at the position of the fish’s maximum girth, *V_R_* was the water velocity at the rear of the flume, and ∈_S_ was the error due to solid blocking, which was calculated as:


(3) \begin{equation*} {\in}_S=\tau \lambda \ {\left({A}_0\ /\ {A}_T\right)}^{A\mathit{\exp}} \end{equation*}

where τ is a dimensionless factor for tunnel cross-sectional shape (0.8), and λ is a factor (coefficient) for the shape of the fish. The shape coefficient was set at 0.5 (i.e. the value for a fish with streamlined shape). Α_0_ is the cross-sectional area of the fish and was calculated as 0.25*G*^2^ π^−1^, where *G* was the maximum girth to the closest millimetre. *A_T_* was the cross-sectional area of the swimming chamber calculated as π r^2^ [radius (*r*) = 120 mm], and the fractional area exponent (*A_exp_*) was 1.5 (Kline *et al.,* 2015).

### Measurements of cardiorespiratory function

Oxygen consumption was measured at rest and at all swimming speeds using intermittent closed respirometry (Sandblom *et al.,* 2014; Rodgers *et al.,* 2016; Svendsen *et al.,* 2016; Killen *et al.,* 2021). The fish’s $\dot{\mathrm{M}}$O_2_ was measured by manually stopping the flow of water into the swim tunnel for a period that did not exceed 10 min, this period shorter at higher swimming speeds to ensure that the oxygen level of the seawater in the tunnel never fell to <85% air saturation. The partial pressure of oxygen (PO_2_) in the swim tunnel was continuously measured using a fibre-optic sensor (Dipping probe) connected to a PreSens O_2_ meter (PreSens Precision Sensing GmBH, Regensburg, Germany), and LabChart v8.1.5 (ADInstruments, Dunedin, New Zealand) was used to calculate the slope of the decrease in PO_2_ after a 2-min wait period. This slope typically had an r^2^ value of >95%. However, two fish at rest had an r^2^ < 0.80, and the average r^2^ value for resting fish was 0.87. However, this is not unusual for resting fish with a low metabolic rate (Chabot *et al.,* 2020). Standard metabolic rate (SMR) was calculated by plotting the relationship between the log of metabolism and swim speed (bL s^-1^) and extrapolating back to a swim speed of 0 bL s^−1^. Absolute aerobic scope (AAS) was calculated as the difference between maximum metabolic rate (MMR) and SMR, while ‘realistic’ absolute aerobic scope (AAS_R_) was calculated using resting metabolic rate (RMR) (Porter and Gamperl, 2023). Background measurements of $\dot{\mathrm{M}}$O_2_ were made after each fish was tested, and these were negligible (<1%), indicating that no substantial microbial respiration was occurring (Rodgers *et al.,* 2016; Svendsen *et al.,* 2016).

Heart rate and cardiac output were recorded at the same time points by connecting the flow probe lead to a Transonic^®^ flow meter, and the signal was amplified and filtered using a data acquisition system (MP160; BIOPAC Systems, Inc., Santa Barbara, CA, USA) and a universal interface module (UIM100A, BIOPAC Systems, Inc.) connected to a computer running AcqKnowledge^®^ software (Version 5.0; BIOPAC Systems, Inc.). Heart rate (*f*_H_; in beats per minute) was measured by counting the systolic peaks during two 30-s intervals while the system was closed for respirometry, and values for cardiac output ($\dot{Q}$; the amount of blood pumped by the heart) were recorded in millilitres per minute per kilogram. This allowed for stroke volume (*V*_S_; the amount of blood pumped per heartbeat) to be calculated as $\dot{Q}$/*f*_H_ (in units of millilitres per kilogram) and blood oxygen extraction to be calculated as $\dot{\mathrm{M}}$O_2_/$\dot{Q}$ (in milligrams O_2_ per millilitre blood). Note: the Transonic^®^ flow probes were calibrated using saline and ~10% haematocrit over a range of temperatures (20–40°C) to ensure that flow values measured in fish at the two temperatures were accurate.

Following the *U*_crit_ test and the 2-h post-exhaustion recovery period, all fish were euthanized using a lethal dose of MS-222 (0.4 g l^−1^), and the heart/ventricle was removed and weighed to determine the relative ventricular mass (RVM), calculated as:


(4) $$ \it \it\begin{equation*} RVM=\left[\left( ventricular\ mass\ / \ fish\ mass\right) \times 100\right] \end{equation*}

Finally, *Q*_10_ values (i.e. the fractional change in a rate over a 10°C range) were calculated as an index of the change in a number of parameters/rates (e.g. *U*_crit_, $\dot{\mathrm{M}}$O_2_, *f*_H_, $\dot{Q}$, *V*_S_ and $\dot{\mathrm{M}}$O_2_/$\dot{Q}$) between 22°C (R1 at T1) and 30°C (R2 at T2) using the following equation:


(5) \begin{equation*} {Q}_{10}={\left(\frac{R2}{R1}\right)}^{\left(\frac{10}{\left(T2-T1\right)}\right)} \end{equation*}

### Statistical analyses

A Rosner’s test [*EnvStats* package in R with ⍺ = 0.05 (Millard, 2013)] and a Grubb’s test [*outliers* package in R with ⍺ = 0.05 (Komsta, 2022)] were used to identify outliers in all datasets prior to statistical analysis. However, no outliers were detected. All data were then tested for assumptions of normality and homogeneity of variance using Shapiro–Wilks and Levene’s tests, respectively (Fox and Weisberg, 2019). A Welch’s two-sample *t*-test was used (*stats* package in R) to examine the effect of the seasonal temperatures on all morphometric, cardiac and metabolic data, and a pairwise *t*-test [*tidytests* package in R (Straforelli, 2023)] was used to compare the resting versus 2-h recovery cardiorespiratory data. All statistical analyses were performed using Rstudio v. 2022.12.0 + 353 with R v. 4.2.3 (R Core Team, 2022), and all data in the text, figures and tables are means ± 1 SEM (unless otherwise specified). The threshold used for determining statistical significance was *P* < 0.05.

## Results

### Morphology, swimming performance and aerobic capacity

There were no significant differences in mean body mass, length, girth or relative ventricular mass between the fish caught in the early fall and winter (Supplementary Table S1).

**Figure 1:**
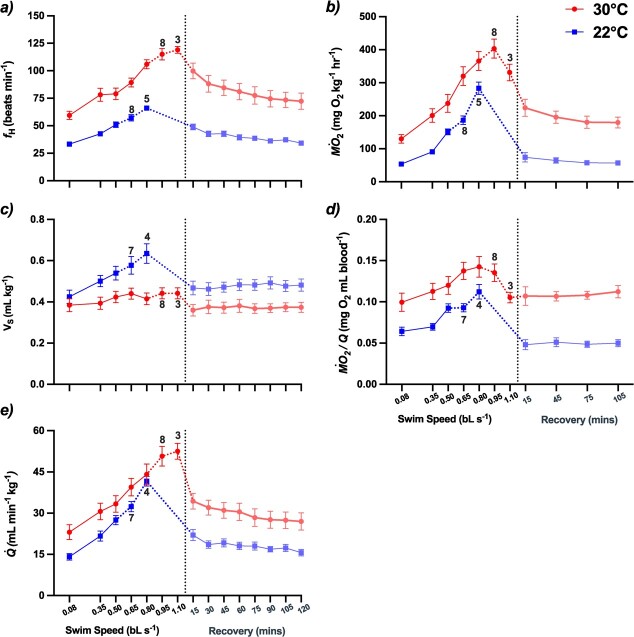
Cardiorespiratory parameters in Nassau grouper at rest (0.08 bL s^−1^), when swum to their critical swimming speed, and then allowed to recover for 2 h at average temperatures in the early fall (30°C; *red* circles) and winter (22°C; *blue squares*). Shown are (**a**) heart rate (*f*_H_), (**b**) oxygen consumption ($\dot{\mathrm{M}}$O_2_), (**c**) stroke volume (*V*_S_), (**d**) blood oxygen extraction ($\dot{\mathrm{M}}$O_2_/$\dot{Q}$) and (**e**) cardiac output ($\dot{Q}$). Values are means ± 1 SEM, with *n* = 9 per group. Numbers above the points indicate when *n* < 9. Note: Swimming velocity values in the figure are not corrected for solid blocking.

The SMR and RMR values for grouper at 30°C were 2.5-fold greater than in the 22°C fish (*P* < 0.001), and the *Q*_10_ values for both of these parameters were quite high [i.e. >3 (Tables 1 and 2)]. The *U*_crit_ of grouper tested at 30°C was significantly greater (by 27%) than that of fish tested at 22°C (Table 1), and this resulted in a *Q*_10_ value over this temperature range of 1.57 (Table 2). As expected based on the *U*_crit_ data, the MMR and AAS_R_ of fish swum at 30°C were ~45 and 37% greater, respectively, than those tested at 22°C. However, there was no significant difference in the ‘realistic’ factorial aerobic scope (FAS_R;_ MMR/RMR) between the two groups, whereas FAS (MMR/SMR) was actually 50% greater in the 22°C fish (Table 1). Following the *U*_crit_ test at 22°C, all of the above parameters returned to pre-test levels after ~105–120 minutes (Fig. 1, Table 3). In contrast, $\dot{\mathrm{M}}$O_2_ was still ~40% higher in grouper tested at 30°C after 2 h of recovery versus before swimming.

**Table 1:** The critical swim speed (*U*_crit_; bL sec^−1^); SMR, RMR and MMR (milligrams O_2_ per kilogram per hour); ‘realistic’ absolute aerobic scope (AAS_R_ and FAS_R_; using RMR) and aerobic scope (AAS and FAS; using SMR); and the resting, maximum and scope values for heart rate (*f*_H_; beats per minute); cardiac output ($\dot{Q}$; millilitres per minute per kilogram); stroke volume (*V*_S_; millilitres per kilogram) and oxygen extraction ($\dot{\mathrm{M}}$O_2_/$\dot{Q}$; milligrams O_2_ per millilitres blood), for Nassau grouper tested at 30 and 22°C

	**22°C**	**30°C**
***U***_**crit**_	0.70 ± 0.04^a^	0.96 ± 0.04^b^
$\dot{\textbf{M}}$ **O_2_**		
SMR RMR MMR AAS FAS AAS_R_ FAS_R_	44.08 ± 4.09^a^ 53.73 ± 3.99^a^ 230.32 ± 24.25^a^ 186.23 ± 23.92^a^ 5.49 ± 0.69^a^ 176.59 ± 23.32^a^ 4.38 ± 0.48^a^	117.71 ± 12.57^b^ 129.68 ± 12.93^b^ 409.40 ± 26.99^b^ 291.69 ± 22.30^b^ 3.63 ± 0.26^a^ 279.72 ± 19.97^b^ 3.27 ± 0.19^b^
***f*_H_**		
Rest Max AS FS	33.3 ± 0.8^a^ 62.4 ± 2.3^a^ 29.1 ± 1.9^a^ 1.87 ± 0.06^a^	58.3 ± 4.2^b^ 118.8 ± 3.4^b^ 60.5 ± 3.9^b^ 2.10 ± 0.14^a^
***V*_S_**		
Rest Max AS FS	0.425 ± 0.032^a^ 0.589 ± 0.039^a^ 0.164 ± 0.021^a^ 1.40 ± 0.06^a^	0.385 ± 0.031^a^ 0.462 ± 0.023^b^ 0.078 ± 0.015^b^ 1.24 ± 0.06^a^
** $\boldsymbol{\dot{Q}}$ **		
Rest Max AS FS	14.14 ± 1.21^a^ 35.84 ± 2.62^a^ 21.71 ± 1.83^a^ 2.58 ± 0.15^a^	23.10 ± 2.73^b^ 51.16 ± 3.36^b^ 28.07 ± 2.00^b^ 2.35 ± 0.18^a^
** $\dot{\textbf{M}}$ O_2_/$\boldsymbol{\dot{Q}}$**		
Rest Max AS FS	0.064 ± 0.005^a^ 0.107 ± 0.006^a^ 0.043 ± 0.008^a^ 1.76 ± 0.18^a^	0.098 ± 0.010^b^ 0.149 ± 0.011^b^ 0.052 ± 0.007^a^ 1.59 ± 0.10^a^

Values for both absolute (AS) and factorial (FS) scope are reported for each cardiac parameter. Values are means ± 1 SEM, with *n* = 9 per group. Dissimilar letters indicate values that are significantly different (*P* < 0.05) between the two test temperatures.

### Cardiac function and oxygen extraction

There was a 2-fold decrease in resting (~58 vs. 33 beats min^−1^; *P* < 0.001) and maximum (119 vs. 62 beats min^−1^; *P* < 0.0001) *f*_H_, and in the absolute scope for *f*_H_ (60 vs. 29 beats min^−1^; *P* < 0.0001), between 30 and 22°C, respectively (*Q*_10_ ~2–2.5; Tables 1 and 2, and Fig. 1a). However, the factorial scope for *f*_H_ was similar (Table 1). In contrast, while *V*_S_ was not significantly different in grouper at rest (0.425 ± 0.032 vs. 0.385 ± 0.031 ml kg^−1^ at 22 vs. 30°C, respectively; *P* = 0.387), this parameter increased by ~38.5% in 22°C fish during the *U*_crit_ test but not in 30°C fish. This resulted in *V*_SMax_ for 22°C fish being 27.5% greater than that measured in 30°C conspecifics (Table 1), and *Q*_10_ values for *V*_SMax_ and *V*_S_ scope <1 (~0.8; Table 2). This disparate increase in *V*_S_ with swimming speed resulted in $\dot{Q}$ values (which were ~40% greater in fish at 30 vs. 22°C at rest) that were similar in both groups at the *U*_crit_ of the 22°C fish. While $\dot{Q}$_Max_ was 1.43-fold greater in 30 vs. 22°C fish (51.16 ± 3.3 vs. 35.84 ± 2.62; *Q*_10_ = 1.6), there was no significant difference in this parameter’s factorial scope (~2.5). Oxygen extraction was significantly (by 35%) greater in 30 versus 22°C grouper at rest, but increased by a similar amount (~1.6- to 1.7-fold; Fig. 1) in both groups during the *U*_crit_ test (*Q*_10_ ~1; Table 2). In contrast to the other cardiorespiratory parameters, which were not different pre- and post-test in 22°C fish, the $\dot{\mathrm{M}}$O_2_/$\dot{Q}$ of this group was actually lower after the fish recovered for 2 h (0.064 ± 0.005 vs. 0.050 ± 0.004 mg O_2_ ml blood^−1^, respectively; Table 3).

## Discussion

As expected, the Nassau grouper had values for SMR, RMR, MMR, AAS and *U*_crit_ that were considerably higher in the early fall (i.e. at 30°C) as compared to during the winter (i.e. at 22°C) (see Table 1 and Fig. 1b). However, (i) the role played by *f*_H_ and *V*_S_ in supporting maximal swimming was quite different at the two temperatures. In 30°C fish, *f*_HMax_ and the scope for *f*_H_ were 119 and 61 beats min^−1^, respectively, and thus, there was no need to increase *V*_S_ during the *U*_crit_ trial. Whereas these values were only 62 and 29 beats min^−1^ in grouper at 22°C, and this required that *V*_S_ increase by 38.5%. (ii) In contrast to *f*_H_ and $\dot{Q}$_,_ there was no temperature effect on the absolute scope of tissue oxygen extraction in Nassau grouper when swum to exhaustion (i.e. *Q*_10_ = 1; Table 2). These data provide key information with regard to the temperature-dependent physiology of an important (and endangered) marine fish species and broaden our understanding of the mechanisms that mediate cardiorespiratory plasticity in fishes and the capacity of this system to support maximum levels of exercise.

**Table 2:** *Q*_10_ values for the effect of seasonal average temperatures (22 vs. 30°C) on resting, maximum and the absolute scope of cardiorespiratory parameters

	*Q*_10_
**SMR**	4.07
**RMR**	3.42
**MMR**	2.50
**AAS**	2.49
***U*_crit_**	1.52
***f*_H_**	
Resting Max Abs. scope	2.06 2.29 2.62
***V*_S_**	
Resting Max Abs. scope	0.85 0.75 0.78
** $\boldsymbol{\dot{Q}}$ **	
Resting Max Abs. scope	1.87 1.60 1.52
** $\dot{\textbf{M}}$ O_2_/$\boldsymbol{\dot{Q}}$**	
Resting Max Abs. scope	1.89 1.57 1.04

### Aerobic capacity and swimming performance

#### Tropical and subtropical marine fishes

Significant knowledge gaps still exist with regard to the metabolic and performance capacity of marine fish species that live in these subtropical and tropical regions/at warm temperatures. The swimming energetics of high-performance pelagic fishes such as tuna, mahi-mahi (*Coryphaena hippurus*), yellowtail kingfish (*Seriola lalandi*) and cobia (*Rachycentron canadum*) have been a primary focus in this area given their relevance for recreational/sport fisheries and aquaculture production (Clark and Seymour, 2006; Dewar and Graham, 1994; Gooding *et al.,* 1981; Heuer *et al.,* 2021; Morgenroth *et al.*, 2022; Nelson *et al.,* 2017). However, it is imperative that data be collected on important (keystone) members of subtropical/tropical reef communities. Reefs are critically important marine ecosystems; the fish and other organisms inhabiting them face imminent threats from climate change (França *et al.,* 2020; Setter *et al.,* 2022), and metabolic capacity (i.e. MMR and aerobic scope) has been strongly linked to a fish’s lifestyle and ecotype (Norin and Clark, 2016; Killen *et al.,* 2017; Fu *et al.,* 2022). Thus, data on the temperature dependence of cardiorespiratory function in pelagic fishes is unlikely to reflect that of many species that inhabit coral reefs (e.g. the Nassau grouper, which is a relatively sedentary ambush predator).

The Nassau grouper had very low values for *U*_crit_ (<1 bL s^−1^) and MMR (~400 mg O_2_ kg^−1^ h^−1^) as compared to other tropical/subtropical species (Table 4). This was not unexpected as low values for these parameters are characteristic of slow-growing benthic fishes (Clark *et al.,* 2013; Norin and Clark, 2016; Killen *et al.,* 2017; Fu *et al.,* 2022), and it has been suggested that ambush predators rely heavily on anaerobic or ‘burst’ swimming and have a low sustained aerobic capacity. Further, our data compare favourably with unpublished data for the gag (*Mycteroperca microlepis*; Kline, 1994) and Nassau (Daugherty, 2021) grouper. For example, MMR in the three studies is within ~10%, and the values for AAS are quite similar when one considers that the fish used in Daugherty (2021) were domesticated (F1) fish and had a lower SMR than in this study, and Kline (1994) also used wild fish within a few weeks of capture (Table 4). In addition, while the *U*_crit_ of Nassau grouper at 30°C was less in our study (0.96 bL s^−1^) as compared to the two unpublished theses (1.48 and 2.0 bL s^−1^ for gag and Nassau grouper, respectively) this is easily explained. First, our fish had undergone surgery so that cardiac variables could also be measured, and Petersen and Gamperl (2010) showed that surgical implantation of blood flow probes reduced the *U*_crit_ of Atlantic cod by 14–30%. Second, in Daugherty (2021), ~25% of the fish tested were termed ‘failures’ and excluded from the study, and thus, the *U*_crit_ that they report is likely an overestimation. Based on the above, we suggest that the *U*_crit_ of wild grouper over the size range in the three studies at 30°C is between 1.25 and 1.5 bL s^−1^. In contrast, however, the maximum metabolic rate that can be achieved in the Nassau grouper is still not known as it has been suggested that ambush predators typically achieve a higher MMR during digestion of large meals in combination with exhaustive recovery (Clark *et al.,* 2013; Fu *et al.,* 2022). That said, freshwater pike (*Esox lucius*) are also ambush predators that eat large meals (up to 10% of their body mass), and MMR after a meal in 1-kg fish is actually less than that following a chase to exhaustion [128 vs. 158 mg O_2_ kg^−1^ h^−1^ (Armstrong *et al.,* 1992)].

While we were anticipating that the MMR and AAS of the Nassau grouper would be much less than top pelagic predators such as the mahi-mahi and yellowfin tuna (*Thunnus albacares*), the large difference in swimming capacity between the grouper and schoolmaster snapper (*Lutjanus apodus*; Malorey *et al.,* unpubl) at similar temperatures was somewhat unexpected (Table 4). Measurements on both species were performed using the same swim tunnel and equipment, and while snapper are considered an ‘opportunistic predator’, subadults and adults of this species co-exist with Nassau grouper on shallow coral reefs/within the same thermal environment (Schneider *et al.,* 2023). That the schoolmaster snapper’s MMR, AAS and *U*_crit_ were ~1.8-, 1.95- and 2.5-fold greater than that of the Nassau grouper, respectively, supports other data that suggest that while temperature is the predominant factor impacting fish biology/physiology (Brett, 1971; also see below), lifestyle (i.e. life history, morphology, feeding behaviour, etc.) also has a major effect (Clark *et al.,* 2013; Norin and Clark, 2016; Killen *et al.,* 2017).

**Table 3:** Cardiorespiratory parameters for Nassau grouper at rest, and 2 h after they completed a critical swimming speed test, at average summer (30°C) and winter (22°C) temperatures. Oxygen consumption ($\dot{\mathrm{M}}$O_2_; milligrams O_2_ per kilogram per hour), heart rate (*f*_H_; beats per minute), cardiac output ($\dot{Q}$; millilitres per minute per kilogram), stroke volume (*V*_S_; millilitres per kilogram) and oxygen extraction ($\dot{\mathrm{M}}$O_2_/$\dot{Q}$; milligrams O_2_ per millilitres blood)

	Rest	After 2 h of recovery
$\dot{\textbf{M}}$ **O_2_**		
22°C 30°C	53.73 ± 3.99 129.68 ± 12.93^a^	56.93 ± 7.83 179.55 ± 16.33^b^
***f*_H_**		
22°C 30°C	33.3 ± 0.8 58.3 ± 4.2	34.2 ± 2.1 72.4 ± 7.4
***V*_S_**		
22°C 30°C	0.425 ± 0.032 0.385 ± 0.031	0.482 ± 0.029 0.374 ± 0.024
$\boldsymbol{\dot{Q}}$		
22°C 30°C	14.14 ± 1.21 23.10 ± 2.73	13.67 ± 3.37 27.00 ± 3.17
$\dot{\textbf{M}}$ **O**_**2**_/$\boldsymbol{\dot{Q}}$		
22°C 30°C	0.064 ± 0.005^a^ 0.098 ± 0.010	0.050 ± 0.004^b^ 0.103 ± 0.013

Values are means ± 1 SEM, with *n* = 9 per group. Values without a letter in common are significantly different at rest versus after 2 h (*P* < 0.05).

#### Grouper metabolism and swimming performance at seasonal temperatures

Many studies on fish show that metabolism is highly sensitive to changes in temperature (see reviews by Farrell *et al.,* 2009 and Schulte, 2015). The Nassau grouper’s SMR and RMR were most affected by seasonal variations in temperature as indicated by the large *Q*_10_ values (~3.4–4.0; Table 1). These *Q*_10_ values are much higher than the 2.2 reported by Hvas *et al.* (2017) for Atlantic salmon acclimated to temperatures ranging from 3 to 23°C, and for wild gag grouper acclimated to 22–30°C (1.5; Kline, 1994). Further, our data for the Nassau grouper do not agree with the thesis of Daugherty (2021), where it was reported that temperature had no effect on the metabolism, AAS or *U*_crit_ of this species. However, these latter data are highly questionable given that temperature influences the rate of biochemical and biological reactions/processes in all ectotherms, and the large number of fish species for which temperature-dependent changes in these parameters are reported. Nonetheless, it does appear based on our study that the Nassau grouper’s respiratory physiology and swimming performance are quite temperature sensitive. Regarding the latter, both this study and Kline (1994) showed that the *U*_crit_ of groupers decreased by ~15–35% between 30 and 22°C.

An interesting observation, afforded by the fact that we are currently conducting experiments on both north temperate and tropical species, is that the SMR and RMR of Atlantic salmon at the lower limit of their thermal range [i.e. 41.7 and 47.5 mg O_2_ kg^−1^ h^−1^, respectively, at 1°C (Porter and Gamperl, 2023)] are comparable to those of Nassau grouper at their lowest seasonal temperature (~22°C; i.e. 44.1 and 53.7 mg O_2_ kg^−1^ h^−1^, respectively). This raises important questions about how some tropical species can have such low metabolic rates. *Q*_10_ values are typically 2–3, and thus, a tropical species would be predicted to have resting metabolic rates at least four times that of salmon at the lower end of their thermal niche (i.e. over a 20°C difference in acclimation/test temperature). Clearly, this is a research area of ‘low-hanging fruit’ and should be further investigated to better understand the cardiorespiratory capacity of marine fishes from different geographic regions.

Starr *et al.* (2007) reported that the average swimming speed of tagged adult Nassau grouper was ~0.7 bL s^−1^ when migrating both to and from shallow spawning sites in the winter. If we consider that our values for *U*_crit_ are underestimated by ~20–30% due to the effects of surgery (Petersen and Gamperl, 2010), then the true *U*_crit_ of the fish in the present study was more likely in the range of 0.85–0.9 bL sec^−1^. Based on these calculations, it would appear that spawning/migrating Nassau grouper are swimming at approximately 80% of their *U*_crit_ at 22–24°C/winter temperatures. At this swimming speed, they would be close to their maximum aerobic capacity (Burgetz *et al.*, 1998), and this suggests that these spawning migrations are metabolically taxing. Interestingly, grouper in this study were only able to fully recover from exhaustive activity within 2 h at the colder temperature (Table 3), and although there were no temperature effects on the absolute scope for tissue oxygen extraction while swimming (*Q*_10_ = 1), values for $\dot{\mathrm{M}}$O_2_/$\dot{Q}$ were surprisingly lower during recovery than at rest in fish at 22°C (Fig. 1d). Collectively, our data suggest that low energetic demands for standard/routine biological functions (i.e. SMR and RMR) at colder temperatures, in addition to a higher factorial aerobic scope for activity at 22 versus 30°C, may facilitate a faster metabolic recovery from maximum aerobic performance and support the hypothesis put forth by Starr *et al.* (2007) that Nassau grouper seek refuge in deeper (colder) thermal environments to better recover from the high metabolic demands of migration and spawning.

#### Cardiac function and the effects of temperature

As with metabolism and swimming capacity, temperature has large effects on the *in vivo* cardiac function of teleost fishes (Eliason and Antitla, 2017). However, this data is almost entirely based on the effects of this important environmental variable on temperate (and some polar) fishes. In fact, to our knowledge, this is the first study to provide comprehensive data on the effects of temperature on the *in vivo* cardiac function of a wild (non-scombrid) tropical marine fish; note, Clark and Seymour (2006) had a limited number of fish (*n* = 2) on which to base temperature-dependent effects. Such information is critical to understanding how climate change will impact subtropical/tropical species, given the important role that cardiac function plays in supporting the fish’s metabolic needs at high temperatures, and that there are now substantial data indicating that cardiac failure is a key factor determining the upper thermal tolerance of this taxon (Ern *et al.,* 2023; Farrell, 2009; Vornanen, 2016 and 2020).

**Table 4:** A comparison of SMR, RMR and MMR (in milligrams O_2_ per kilogram per hour), absolute aerobic scope (AAS; calculated as MMR−SMR), factorial aerobic scope (FAS; calculated as MMR/SMR), and critical swim speed (*U*_crit_; bL per second) at ≥25°C for tropical/subtropical marine fishes ranging from active pelagic predators to low-energy benthic ambush predators

Species	Temp.	~ Mass (g)	SMR	MMR	AAS	FAS	U_crit_ (bL s^−1^)	Lifestyle
Yellowfin tuna (*T. albacares*)^1^	25°C	2200	253.0	2530.0 (estimate)	2277	10.00	2.1^*^	Pelagic high performer/active forager
Mahi-mahi (*C. hippurus*)^3^	28°C	30–40	452.5	1685.8	1122.4	3.73	5.46	Pelagic/deep open ocean top predator
Cobia (*R. canadum*)^2^	26°C	300–400	152.3	1017.0	864.8	6.68	2.01	Pelagic/coastal opportunistic predator
Schoolmaster snapper (*L. apodus*)^4†^	29°C	300	169.2	737.7	568.5	4.41	2.42	Mangrove/reef opportunistic predator
Australasian snapper (*Chrysophrys auratus*)^5^	25°C	115	175.3	579.0	403.7	3.30		Benthopelagic, opportunistic predator
Gag grouper (*M. microlepis*)^6†^	30°C	1800–1950	95.6	430.0	334.4	4.50	1.48	Benthic reef ambush predator
Nassau grouper (*E. striatus*)^7†^	30°C	750–800	62.8	453.3	390.5	7.22^**^	2.0	Benthic reef ambush predator
Nassau grouper (*E. striatus*)^8^	30°C	800–1000	117.7	409.4	291.7	3.50	0.96	Benthic reef ambush predator

1) Dewar and Graham, 1994; 2) Nelson *et al.,* 2017; 3) Heuer *et al.,* 2021; 4) Malorey *et al., in prep*; 5) Bowering *et al.,* 2023 – MMR measured after chase; 6) Kline, 1994; 7) Daugherty, 2021; 8) present study. Single asterisks (^*^) indicate if swim speed (U) was not their critical swim speed, a dagger (^†^) indicates unpublished data, and double asterisks (^**^) indicates that MMR and AS are likely overestimates as poor swimmers (~25% of fish tested) were excluded from the study.

As expected, based on the grouper’s SMR and RMR, they also had low values for resting *f*_H_ and $\dot{Q\ }$at 22°C (~33 beats min^−1^ and 14 ml min^−1^ kg^−1^), as compared to more active species such as the cobia [80 beats min^−1^ and 49.5 ml min^−1^ kg^−1^ at ~25°C (Nelson *et al.,* 2017)] and yellowtail kingfish [~95–100 beats min^−1^ and 60 ml min^−1^ kg^−1^ at ~22–20°C; Clark and Seymour, 2006; Morgenroth *et al.,* 2022). Further, our resting values at 30°C were considerably lower than for the schoolmaster snapper (Sandrelli *et al.,* in prep) at 31°C (58.3 vs. 100 beats min^−1^ and 23 vs. 40 ml min^−1^ kg^−1^, respectively). Interestingly, the modulation of cardiac function and $\dot{\mathrm{M}}$O_2_/$\dot{Q}$ in exercised grouper was also very different as compared to cobia and yellowtail kingfish. In the grouper at 22°C, $\dot{Q}$ increased by ~2.5-fold due to ~1.9- and 1.4-fold increases in *f*_H_ and *V*_S_, respectively (while $\dot{\mathrm{M}}$O_2_/$\dot{Q}$ only increased by ~75%) (Table 1). In contrast, in the cobia and yellowtail, $\dot{Q}$ only increased by 35–55% and $\dot{\mathrm{M}}$O_2_/$\dot{Q}$ increased by an astonishing 4- to 5-fold. This is despite similar values for resting $\dot{\mathrm{M}}$O_2_/$\dot{Q}$, and thus based on the limited data, it appears that active tropical/subtropical pelagic fishes may be quite different in how they meet the metabolic demands of exercise as compared to sedentary reef fishes. This will obviously require data on a wider range of tropical fish species inhabiting different areas, and such studies are planned for the near future.

The *Q*_10_ value for resting $\dot{Q}$ between 22 and 30°C was slightly <2 (1.87), and while *f*_H_ had a *Q*_10_ value of 2.06, that for *V*_S_ was only 0.85 (i.e. it decreased by ~10%) (Tables 1 and 2). Interestingly, a similar trend was observed when the data for maximum cardiac parameters were compared. For example, while *f*_HMax_ approximately doubled at both temperatures during the *U*_crit_ test, both *V*_SMax_ and the scope for *V*_SMax_ were significantly greater (by ~1.27- and 2.1-fold, respectively) in fish swum at 22°C. This is likely because the scope for *f*_H_ was in fact much lower at 22 versus 30°C (~29 vs. 61 beats min^−1^, respectively) and the *Q*_10_ value for the absolute scope for $\dot{\mathrm{M}}$O_2_/$\dot{Q}$ was ~1. Overall, these data suggest that *V*_S_ is more important for achieving $\dot{Q}$_Max_ in the Nassau grouper at cold versus warm temperatures. A greater *V*_S_ due to cardiac hypertrophy/hyperplasia (enlarged hearts) has been documented in several temperate fishes acclimated to cold temperatures (Driedzic *et al.,* 1996; Klaiman *et al.,* 2011; Porter and Gamperl, 2023), and while this study did not acclimate animals, *per se*, it is clear that grouper at 22°C exhibited a higher *V*_SMax_ without a significant increase in RVM (Table 1 and Supplementary Table S1, respectively). Collectively, these *in vivo* data, and that of Porter and Gamperl (2023) and Korsmeyer *et al.* (1997), provide support for the hypothesis of Gamperl *et al.* (2022) that, based on the kinetics of ventricular muscle contraction, *V*_S_ must play an increased role in situations where tissue oxygen demand (and $\dot{Q}$) rises at cold temperatures and emphasize the importance of cardiac plasticity (i.e. the modulation of different cardiovascular mechanisms) in fish for maintaining adequate oxygen delivery/performance with changes in ambient temperature.

## Conclusions and Perspectives

Overall, this work provides novel information on the cardiorespiratory capacity and swimming performance of wild Nassau grouper and has important implications for the development of conservation and management strategies for vulnerable fish populations. For example, our work reveals that this species experiences large reductions in standard and resting metabolic rates at seasonally low temperatures, and thus provides evidence to support the hypothesis of Starr *et al.* (2007) that ‘cold’ thermal environments (deeper depths) may be advantageous for grouper to recover from the large metabolic expenditures associated with migrating and spawning during the winter months. Currently, there are several regulations prohibiting the capture of Nassau grouper in The Bahamas (i.e. no fish <3 lbs) in addition to seasonal closures (1 December through 28 February) during their spawning season (Breef, 2020). However, it might also be prudent to incorporate depth limits after the spawning season into fisheries management plans whose goal is to conserve the species.

This study also reveals two interesting pieces of information about the temperature-dependent cardiorespiratory physiology of this species that have important implications for our understanding of how fish modulate cardiac function when they swim at different temperatures. The first important finding was that temperature had a significant impact on the contributions of *f*_H_ and *V*_S_ to the cardiac output and oxygen consumption of the grouper when swimming. Specifically, just like for the Atlantic salmon (Porter and Gamperl, 2023), at temperatures near the lower end of the grouper’s thermal niche, the capacity to increase *f*_H_ is limited, and *V*_S_ plays a greater role in the increase in $\dot{Q}$ associated with exercise. Second, in more active tropical species, the predominant contributor to exercise-induced increases in $\dot{\mathrm{M}}$O_2_ (Morgenroth *et al.,* 2022; Nelson *et al.,* 2017) is not *f*_H_ but oxygen extraction. The latter only increased by ~75% in the Nassau grouper when swum to their *U*_crit_, and its contribution (absolute and factorial scope) was not temperature-dependent. These are interesting findings from the perspective of understanding how temperature limits the cardiorespiratory function of fishes and interspecific differences in how fish meet the increased oxygen demands of swimming.

## Supplementary Material

Web_Material_coad086
