# Supplementary material for: Seasonal temperatures in South Eleuthera, The Bahamas, have considerable impacts on the cardiorespiratory function and swimming performance of Nassau grouper (Epinephelus striatus)
Source: Conserv Physiol. 2023 Dec 5;11(1):coad086. doi: 10.1093/conphys/coad086 (PMC10699750; doi:10.1093/conphys/coad086)
Supplement: Web_Material_coad086 [file web_material_coad086.pdf]

Seasonal Temperatures in South Eleuthera, The Bahamas, Have Considerable Impacts on the Cardiorespiratory Function and Swimming Performance of Nassau Grouper (*Epinephelus striatus*)

E.S. Porter* and A.K Gamperl

Supplementary Material

Dept. of Ocean Sciences, Memorial University of Newfoundland and Labrador, St. John's, NL. Canada. A1C 5S7.

* Corresponding Author:

Ms. Emma Porter
Dept. of Ocean Sciences,
Memorial University of Newfoundland and Labrador,
St. John's, NL. A1C 5S7.
Canada.

E-mail: esporter@mun.ca
Phone 1-613-453-5714

Supplemental Table 1. Morphometric data (mean ± 1 s.e.m.) for fish at average summer and winter temperatures. None of the parameters were significantly different.

Parameter	Winter (22°C)	Summer (30°C)	p-value
Weight (g)	794.1 ± 37.6	932.6 ± 78.9	0.140
Length (cm)	38.2 ± 0.8	39.0 ± 1.6	0.655
Condition Factor (k)	1.43 ± 0.04	1.47 ± 0.04	0.454
Girth (cm)	25.7 ± 0.4	27.8 ± 1.0	0.078
RVM	0.0534 ± 0.0024	0.0499 ± 0.0024	0.336

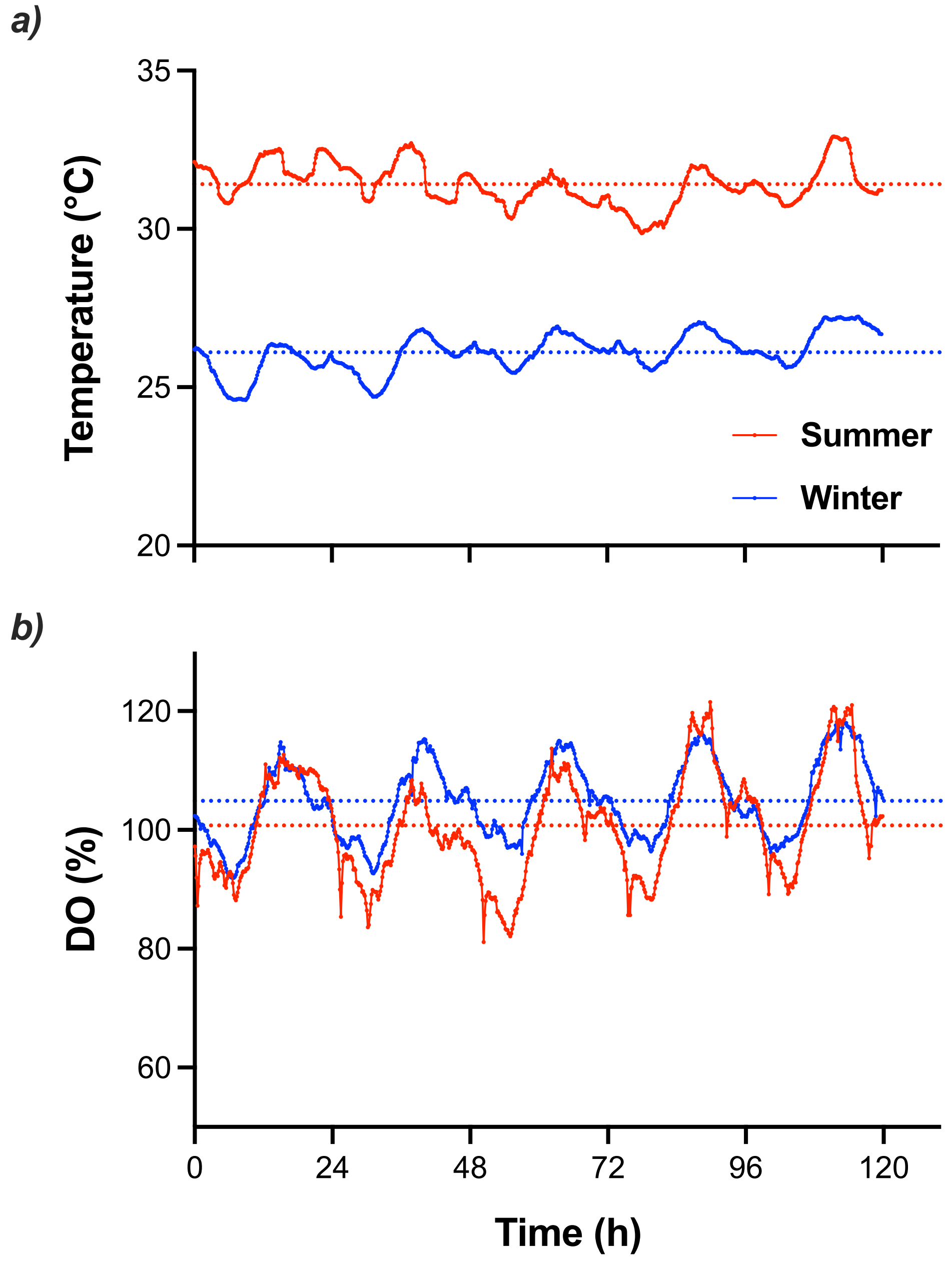

Supplementary Figure 1. The *a)* temperature (°C) and *b)* dissolved oxygen (DO; %) profiles recorded by miniDOT Clear® loggers deployed at a patch reef (5-7 m deep) during the winter (*blue*; February 25th to March 1st) and summer (*red;* July 12-16th, 2023) in South Eleuthera, The Bahamas. Dotted lines represent the seasonal average temperature and DO recorded over 120 hours starting at midnight (0 h).
